# Production of Nematicidal Pinosylvin Stilbenes in Cell Suspension Cultures of *Pinus koraiensis* by Fungal Elicitation

**DOI:** 10.3390/plants11212933

**Published:** 2022-10-31

**Authors:** Yi Rae Kim, Jung Yeon Han, Yong Eui Choi

**Affiliations:** Department of Forest Resources, College of Forest and Environmental Sciences, Kangwon National University, Chuncheon 200-701, Korea

**Keywords:** *Pinus koraiensis*, pinosylvin stilbenes, pinosylvin monomethylether, dihydropinosylvin monomethylether, callus culture, fungal elicitor treatment

## Abstract

Pinosylvin stilbenes are natural phenolic compounds found in the Pinaceae family and act as phytoalexins. Some pinosylvin stilbenes have strong nematicidal activity against pine wood nematodes (PWNs: *Bursaphelenchus xylophilus*). Here, we established the efficient production of two pinosylvin stilbenes, dihydropinosylvin monomethylether (DPME) and pinosylvin monomethylether (PME), by cell suspension culture of *Pinus koraiensis* after fungal elicitation. DPME and PME were found in small amounts (less than 40 µg/g DW) in the stem bark and leaves of *P. koraiensis* plants. Cell suspension cultures were established from the cultures of calli derived from mature zygotic embryos of *P. koraiensis* in 1/2 Litvay medium containing 2.2 μM 2,4-D and 2.2 μM BA. Two types of fungal elicitors, fungal cell extract (CE) and fungal medium filtrate (MF), were prepared from three species of fungi (*Penicillium chrysogenum, P. pinophilum*, and *P. roquefortii*). CE and MF treatments strongly stimulated the production of PME and DPME in cultured cells. The production of PME in suspension cells of *P. chrysogenum, P. pinophilum*, and *P. roquefortii* MF treatments after 3 days was 5734 µg/g DW, 4051 µg/g DW, and 6724 µg/g DW, respectively. Pinosylvin synthase (*PkSTS*) and pinosylvin *O*-methyltransferase (*PkPMT*) are key genes in DPME and PME biosynthesis. qPCR analysis revealed that the expression of the *PkSTS* and *PkPMT* in cultured cells was highly enhanced after fungal elicitor treatment. The cell extracts after MF treatment resulted in 92.5 ± 7.8% immobilization of the adult PWNs and 63.7 ± 3.5% immobilization of the juvenile PWNs within 24 h. However, control cell extracts without MF treatment showed 11.3 ± 1.4% nematicidal activity against adult PWNs. Our results suggest that pinosylvin stilbenes can be produced from the cell culture of *P. koraiensis* after fungal elicitor treatment and can be used as nematicidal compounds against PWNs.

## 1. Introduction

Pine wilt disease (PWD) caused by pine wood nematodes (PWNs; *Bursaphelenchus xylophilus*) is the most serious disease of pine plants [1,2]. Pine destruction by the spread of PWD is a major economic, ecological, and environmental concern worldwide [3]. The spread of PWD is the result of complex interactions among PWNs, insect vectors (*Monochamus* species), and host trees [1,4,5]. The current methods of PWD control mainly rely on trunk injection of nematicides such as abamectin and emamectin, which are macrocyclic lactones [6,7]. The invention of new nematicidal compounds is one of the important challenges to seek alternative chemicals to combat PWNs. Plant extracts such as essential oils and volatile compounds showed high activities on PWNs [8,9].

Stilbenoids are naturally occurring phenolic compounds found in limited distribution in the Pinaceae and Vitaceae families together with other families, such as Dipterocarpaceae, Gnetaceae and Fabaceae [10]. Stilbenoids are known as phytoalexins and act as antimicrobial compounds to protect against fungal and bacterial infections [11]. Stilbenoids in pine plants are mainly the pinosylvin-type, which are particularly rich in heartwood, preventing wood tissues from decaying by fungi [12,13]. The amount of pinosylvin stilbenes in the sapwood and needles of pine plants is very low or not present, and abiotic and/or biotic treatments are required to stimulate the accumulation of the compounds [14]. It has been reported that some of pinosylvin stilbenes have high nematicidal activity against PWNs [15,16,17]. Hwang et al. [16] reported that the accumulation of the two types of pinosylvin stilbenes, DPME and PME, was highly enhanced by PWN infection and involved in the PWN resistance of *Pinus strobus* trees. *Pinus rigida* plants have a strong resistance against PWNs [17]. Two pinosylvin stilbenes (trans-3,5-dimethoxystilbene and cis-3,5-dimethoxystilbene) were accumulated in *P. rigida* resin [17]. cis-3,5-Dimethoxystilbene showed high nematicidal activity, but other compounds did not [17]. These reports indicate that pinosylvin stilbenes may be used as nematicidal compounds.

Plant cell culture is useful a technology for producing high-value secondary metabolites under in vitro controlled environments [18,19]. Resveratrol (*trans*-3,5,4′-trihydroxystilbene) is a well-known stilbenoid that exists in various plants, including grapes, peanuts, and berry fruits [20]. Production of resveratrol and its derivatives via cell suspension culture has been reported in many articles, particularly using grapevine cell cultures [21]. Elicitation is effective for the enhancement of resveratrol production in grapevine cell culture [21]. Only two articles reported the production of pinosylvin-type stilbenes in cell suspension cultures of *Pinus sylvestris* [22] and *P. strobus* [23]. Fungal elicitor treatment prepared from the pine needle fungus (*Lophodermium seditiosum*) resulted in strong accumulation of pinosylvin stilbenes in *P. sylvestris* cell suspension cultures [22]. Koo et al. [23] reported that the production of pinosylvin stilbenes in callus culture of *P. strobus* is associated with the browning process of calli.

*Penicillium* is a genus of ascomycetous fungi and is the most widespread fungi in nature. *Penicillium* is involved in food spoilage and in food and drug production. It has been reported that the fungus *Penicillium* is commonly distributed on PWNs and their vectors and hosts [24].

Here, we established an in vitro callus and cell suspension culture of *P. koraiensis* and investigated the enhanced accumulation of pinosylvin stilbenes in cultured cells by the treatment of fungal elicitors prepared from three *Penicillium* species. The ethanolic extracts from the *P. koraiensis* cell suspension after fungal elicitor treatment had strong nematicidal activity against PWNs.

## 2. Results

### 2.1. Content of Pinosylvin Stilbenes (DPME and PME) in Stem Bark in P. koraiensis

**Figure 1:**
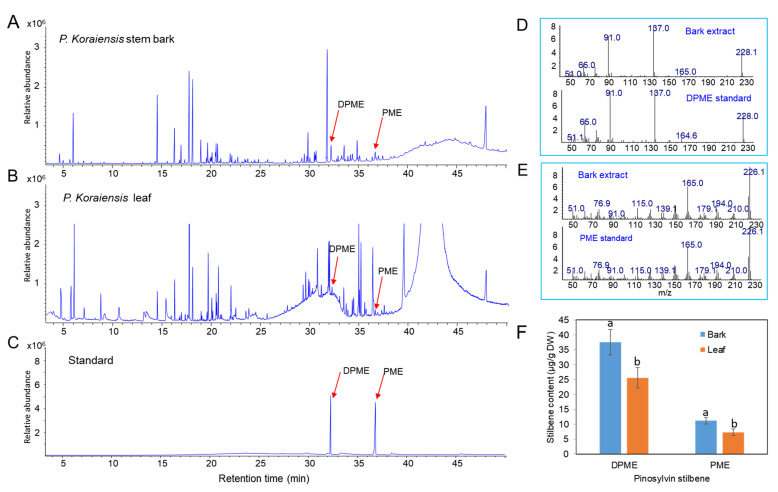
Analysis of DPME and PME in extracts of stem barks and needles of *P. koraiensis* plants by GC–MS analysis. (**A**) GC chromatogram of stem bark extracts. (**B**) GC chromatogram of needle extracts. (**C**) GC chromatogram of DPME and PME standards. (**D**) Mass fractions of the DPME peak (32.2 min) detected in stem bark extracts and DPME standards. (**E**) Mass fractions of the PME peak (36.6 min) detected in needle extracts and DPME standards. (**F**) Content of DPME and PME in stem barks and needles of *P. koraiensis* plants. Different letters above the bars indicate significantly different values (*p* < 0.05), calculated using one-way ANOVA followed by *Duncan’s post hoc analysis*.

Methanol extracts from stem bark and leaves of *P. koraiensis* were used to identify and examine the content of pinosylvin stilbenes (DPME and PME) by GC/MS. DPME and PME were detected at retention times of 32.3 and 36.7 min, respectively (Figure 1A,C). The presence of the two stilbenes was confirmed by comparison of the mass fraction patterns of DPME and PME standards (Figure 1D,E). The contents of both DPME and PME were richer in stem bark than in leaves (Figure 1F). DPME was present in a higher amount than PME in both stem bark and leaves (Figure 1F). The contents of DPME and PME in stem bark were 37.5 µg/g DW and 11.2 µg/g DW, respectively (Figure 1F). The contents of DPME and PME in needles were 25.6 µg/g DW and 7.3 µg/g DW, respectively (Figure 1F).

### 2.2. Induction and Proliferation of P. koraiensis Calli

**Figure 2:**
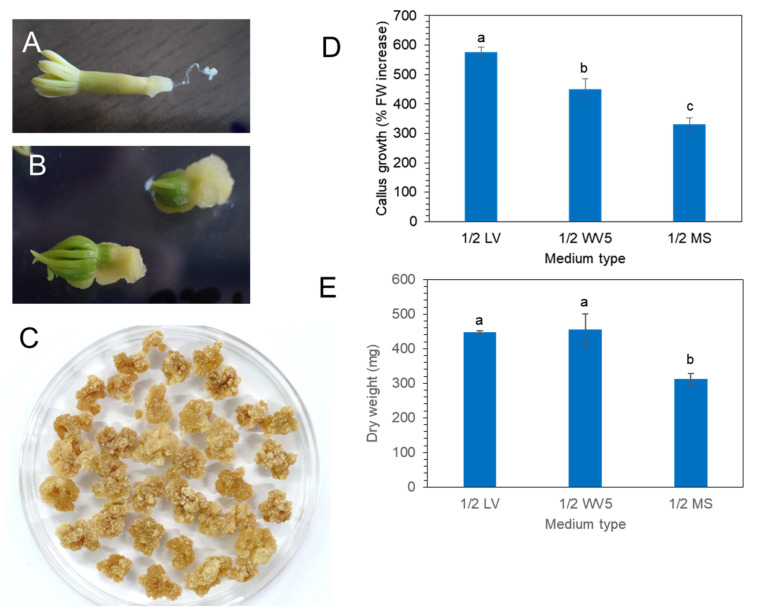
Callus induction and proliferation from mature zygotic embryos on three different media with 4.4 μM 2,4-D and 2.2 μM BA. (**A**) Mature zygotic embryos of *P. koraiensis*. (**B**). Callus induction on radicle portion of zygotic embryos after two weeks. (**C**) Proliferated calli after consecutive subculture on new 1/2 LV medium with 4.4 μM 2,4-D and 2.2 μM BA. (**D**) Fresh weight of calli on different media (1/2 LV, 1.2 WV5, and 1/2 MS) with 4.4 μM 2,4-D and 2.2 μM BA after 3 weeks of culture. (**E**) Dry weight of calli on different media (1/2 LV, 1.2 WV5, and 1/2 MS) with 4.4 μM 2,4-D and 2.2 μM BA after 3 weeks of culture. Different letters above the bars indicate significantly different values (*p* < 0.05), calculated using one-way ANOVA followed by *Duncan’s post hoc analysis*.

Mature zygotic embryos (Figure 2A) of *P. koraiensis* were cultured on 1/2 LV medium with 4.4 μM 2,4-D and 2.2 μM BA. Callus was induced from zygotic embryos after 3 weeks of culture (Figure 2B). After subculture of the callus onto the same medium at two-week intervals, friable callus masses were obtained (Figure 2C).

Calli were transferred onto three different media (1/2 MS, 1/2 LV, or 1/2 WV5) with 4.4 μM 2,4-D and 2.2 μM BA to evaluate the best medium for callus proliferation. Fresh weight and dry weight were calculated after 3 weeks of culture (Figure 2D,E). The fresh weight of callus showed that the 1/2 LV medium was the most effective for callus growth compared to 1/2 MS and 1/2 WV5 (Figure 2D). However, the dry weight of callus showed no significant difference between the 1/2 LV and 1/2 WV5 medium. The proliferation of calli on the 1/2 MS medium was not superior to that on the 1/2 LV and 1/2 WV5 medium (Figure 2E).

### 2.3. Cell Suspension Culture

**Figure 3:**
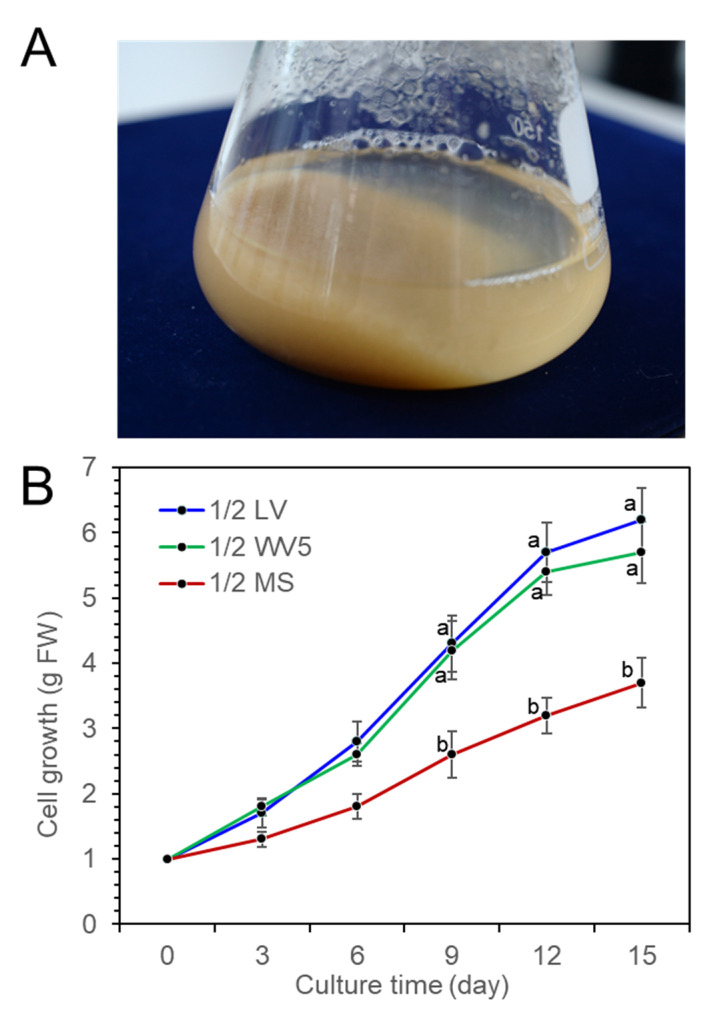
Proliferation of *P. koraiensis* cells in three different media (1/2 LV, 1.2 WV5, and 1/2 MS) containing 2.2 μM 2,4-D with 2.2 μM BA after 15 days of culture. (**A**) Cell suspension in a 250-ml flask containing 1/2 LV liquid medium with 2.2 μM 2,4-D with 2.2 μM BA after 15 days of culture. (**B**) Growth of cells (fresh weight) in three different media (1/2 LV, 1.2 WV5, and 1/2 MS) containing 2.2 μM 2,4-D and 2.2 μM BA during 15 days of culture. One gram of initial fresh weight of cells was inoculated in liquid media. Error lines represent ±SE of the mean of three independent experiments, each performed in technical triplicates. Different letters above the bars indicate significantly different values (*p* < 0.05), calculated using one-way ANOVA followed by *Duncan’s post hoc analysis*.

Fine cell suspension culture was achieved by a shake flask culture of *P. koraiensis* calli in 1/2 LV liquid medium with 4.4 μM 2,4-D and 2.2 μM BA (Figure 3A). To investigate the proliferation of suspension cells in liquid medium, 1 g of cells was transferred into three different liquid media (1/2 LV, 1/2 WV5, and 1/2 MS) with 2.2 μM BA, 2.2 μM 2,4-D and 20 g/L sucrose in 250 mL flasks containing 75 mL medium. The fresh weight of the cell mass was measured at 3-day intervals for 15 days of culture. Proliferation of cells was actively achieved in both the 1/2 LV and 1/2 WV5 liquid media compared to the 1/2 MS liquid medium. There was no significant difference in cell growth between the 1/2 LV and 1/2 WV5 liquid media, but the 1/2 MS medium was less effective for cell growth than the 1/2 LV and 1/2 WV5 media (Figure 3B).

### 2.4. Enhanced Production of DPME and PME in Cell Suspension by Fungal Elicitor Treatment

**Figure 4:**
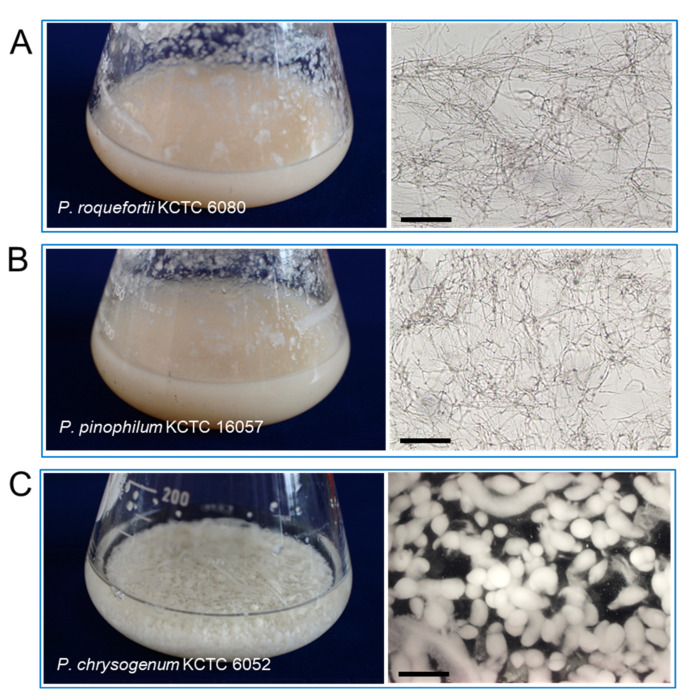
Photos and microscope observation of fungal mycelia grown in a shake flask culture after 2 weeks of culture in PDA liquid medium. (**A**) Photo of *P. roquefortii* mycelia (left) constituted with homogenous and filamentous mycelia (right) by light microscopy. (**B**) Photo of *P. pinophilum* mycelia (left) constituted with homogenous and filamentous mycelia (right) by light microscopy. (**C**) Photo of *P. chrysogenum* mycelia (left) constituted with spherical mycelium pellets (right) by dissecting microscope (right). Bars in (**A**,**C**) = 50 μm, (**B**) = 700 μm.

The *P. koraiensis* cell suspension culture was maintained by shaking the flask culture in 1/2 LV liquid medium with 2.2 μM 2,4-D with 2.2 μM BA. Three species of fungus, *P. chrysogenum* KCTC 6052, *P. pinophilum* KCTC 16057, and *P. roquefortii* KCTC 6080, were grown in PDA medium for 2 weeks (Figure 4A–C). Mycelium growth in *P. pinophilum* and *P. roquefortii* was homogeneous and filamentous (Figure 4A,B), but mycelia of *P. chrysogenum* were grown in the form of small spherical or spheroidal pellets (Figure 4C).

**Figure 5:**
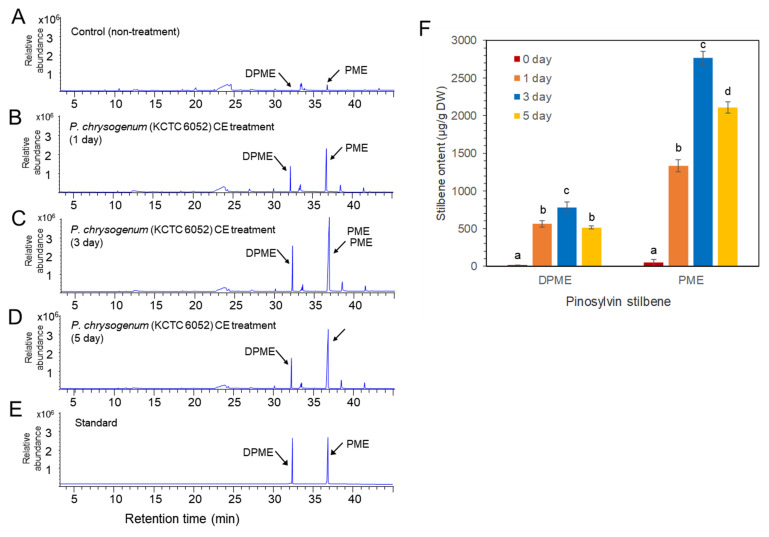
GC/MS analysis of DPME and PME production in cultured cells of *P. koraiensis* by fungal elicitor (*P. chrysogenus* MF) treatment. (**A**) GC chromatogram of extracts from control cells without MF treatment. (**B**–**D**) GC chromatogram of extracts from cultured cells after 1 day (**B**), 3 days (**C**), and 5 days of MF treatment. (**E**) GC chromatogram of authentic standards of DPME and PME. (**F**) Content of DPME and PME in cultured cells during 5 days of culture after MF treatment. Different letters above the bars indicate significantly different values (*p* < 0.05), calculated using one-way ANOVA followed by *Duncan’s post hoc analysis*.

Two types of fungal elicitors, fungal medium filtrate (MF) and fungal cell extract (CE), were prepared from the fungal culture. The CE and MF elicitors were added to a liquid medium after 10 days of cell suspension culture, and the concentration of both CE and MF fungal elicitors was adjusted to 2% (*v*/*v*) in liquid medium. The accumulation of DPME and PME in *P. koraiensis* cells after *P. chrysogenum* MF treatment was monitored at 0, 1, 3, and 5 days of culture. In the control without MF treatment, the accumulation of DPME and PME in cells was 17 ± 1.2 µg/g DW and 47 ± 2.4 µg/g DW, respectively (Figure 5A). The accumulation of both DPME and PME was significantly increased by MF treatment and showed a peak at 3 days of MF treatment (Figure 5B–D). The DPME and PME compounds in *P. koraiensis* cells were identified by standard compounds (Figure 5E). At three days of MF treatment, the DPME content showed a 10-fold increase, and the PME content showed a 97-fold increase compared to the control without MF treatment (Figure 5F). In all treatments, even the control, the content of PME in *P. koraiensis* cells was higher than that of DPME (Figure 5E).

**Figure 6:**
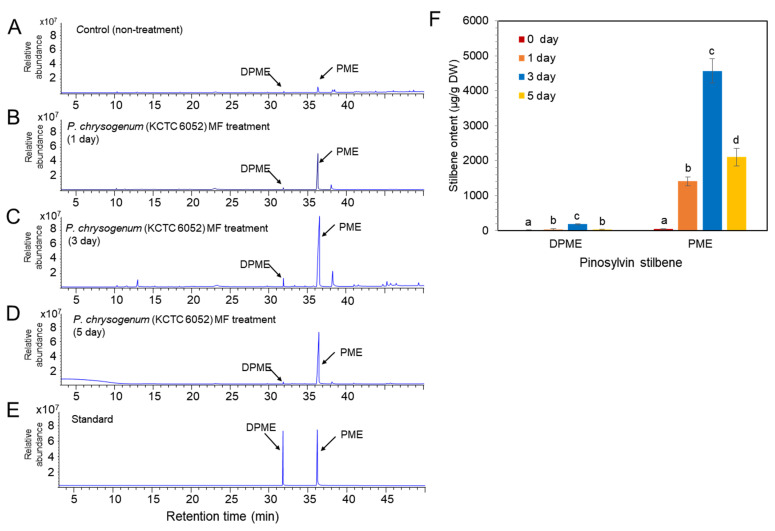
GC/MS analysis of DPME and PME amounts in cultured cells of *P. koraiensis* by fungal elicitor (*P. chrysogenus* CE) treatment. (**A**) GC chromatogram of cell extracts from the control without MF treatment. (**B**–**D**) GC chromatogram of cell extracts after 1 day (**B**), 3 days (**C**), and 5 days (**D**) of MF treatment. (**E**) GC chromatogram of authentic standards of DPME and PME. (**F**) Content of DPME and PME in cultured cells during 5 days of culture after CE treatment. Different letters above the bars indicate significantly different values (*p* < 0.05), calculated using one-way ANOVA followed by *Duncan’s post hoc analysis*.

Similar to MF treatment, *P. chrysogenum* CE treatment resulted in enhanced accumulation of DPME and PME in *P. koraiensis* cells compared to the control (Figure 6). The GC chromatogram and the contents of DPME and PME showed peaks at 3 days of CE treatment (Figure 6). The DPME and PME compounds in *P. koraiensis* cells were identified by comparison of standard compounds (Figure 6D). At three days of CE treatment, the DPME content showed a 37.7-fold increase, and the PME content showed a 56.4-fold increase compared to the control without CE treatment (Figure 6F). Interestingly, DPME accumulation by CE treatment was highly effective compared to MF treatment because the content of DPME (Figure 6E) in CE-treated *P. koraiensis* cells was much higher than that (Figure 5E) in MF-treated *P. koraiensis* cells (Figure 6F). However, the accumulation of PME (Figure 6E) in the CE treatment was lower than that in the MF treatment (Figure 5F).

**Figure 7:**
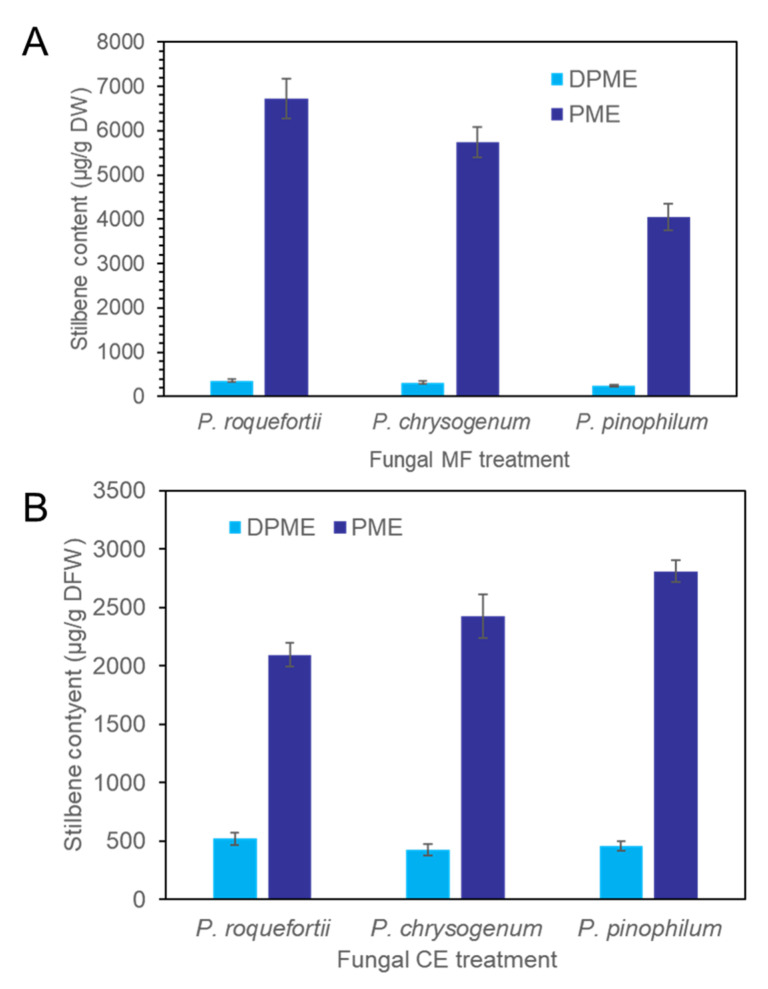
Accumulation of DPME and PME in *P. koraiensis* cells after fungal elicitor treatment. (**A**) Content of DPME and PME in *P. koraiensis* cells after MF treatment of *P. roquefortii*, *P. chrysogenum*, and *P. pinophilum* for 3 days. (**B**) Content of DPME and PME in *P. koraiensis* cells after CE treatment of *P. roquefortii*, *P. chrysogenum*, and *P. pinophilum* for 3 days.

CE and MF solution were prepared from the three species of fungi, *P. chrysogenum, P. pinophilum*, and *P. roquefortii*. The quantitative order of both DPME and PME accumulation in *P. koraiensis* cells after 3 days of MF treatments was *P. roquefortii > P. chrysogenum > P. pinophilum* (Figure 7A). The production of PME in suspension cells after 3 days of *P. chrysogenum, P. pinophilum*, and *P. roquefortii* MF treatments was 5734 µg/g DW, 4051 µg/g DW, and 6724 µg/g DW, respectively. The quantitative order of both DPME and PME accumulation in *P. koraiensis* cells after 3 days of CE treatments was *P. pinophilum > P. chrysogenum > P. roquefortii* (Figure 7B).

### 2.5. DPME and PME Accumulation by Prolonged Culture of Calli

**Figure 8:**
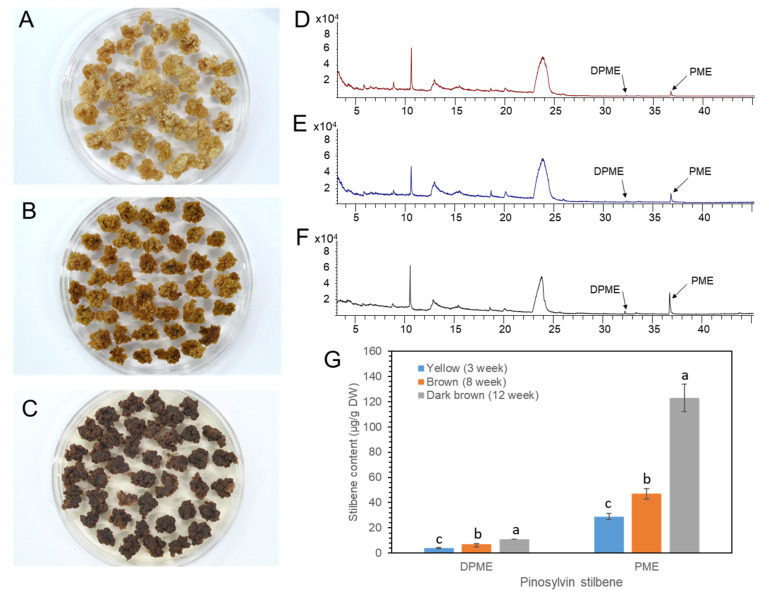
Ageing of *P. koraiensis* callus by prolonged culture and accumulation of DPME and PME in differently aged callus. (**A**–**C**) Photos of yellow (**A**), brown (**B**), and dark brown (**C**) calli after 3 weeks, 8 weeks, and 12 weeks of culture, respectively. (**D**–**F**) GC chromatogram of callus extracts from yellow (**D**), brown (**E**), and dark brown (**F**) calli. (**G**) Content of DPME and PME in calli with different ages. The analysis results are presented as the means ± SEs of three independent experiments, each performed in technical triplicates. Different letters above the bars indicate significantly different values (*p* < 0.05), calculated using one-way ANOVA followed by *Duncan’s post hoc analysis*.

We previously reported that the DPME and PME accumulation in *P. strobus* calli was highly increased by callus ageing [23]. *P. koraiensis* callus was transferred onto 1/2 LV medium with 4.4 μM 2,4-D and 2.2 μM BA and maintained without subculture until 3 months. The color of the calli turned pale yellow after 1 month (Figure 8A), brown after 2 months (Figure 8B), and dark brown after 3 months (Figure 8C).

DPEM and PME accumulation was monitored in MeOH extracts from the calli with different levels of browning. Both DPME and PME were detected in a small amount in one-month-old calli (Figure 8D). DPME and PME accumulations in calli were enhanced as the culture time proceeded to two and three months (Figure 8E,F). The amounts of DPME and PME in three-month-old calli with dark brown colors were 11 µg/g DW and 123 µg/g DW, respectively (Figure 8G).

### 2.6. qPCR Analysis of PsSTS and PsPMT Genes

**Figure 9:**
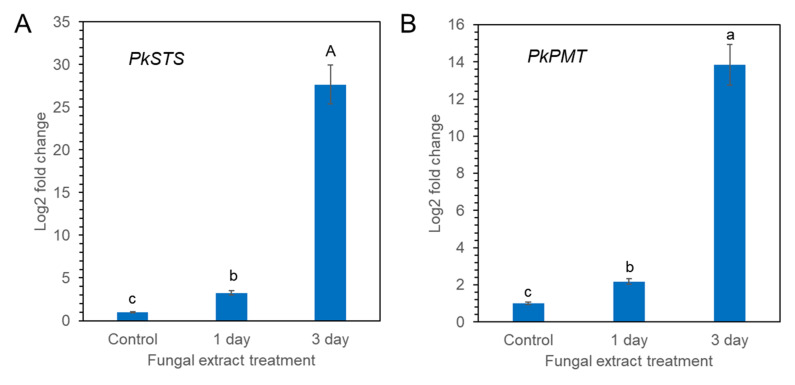
qPCR analysis of *PkSTS* (**A**) and *PkPMT* (**B**) genes in *P. koraiensis* callus after fungal elicitor (*P. chrysogenus* MF) treatment. The expression data were normalized to β-actin. The vertical bars represent the mean ± SE based on three biological replicates. Different letters above the error bars indicate significantly different values (*p* < 0.05), calculated using one-way ANOVA followed by *Duncan’s post hoc analysis*.

Pinosylvin synthase (STS) and pinosylvin *O*-methyltransferase (PMT) are key enzymes involved in pinosylvin stilbene biosynthesis [25,26]. *PkSTS* and *PkPMT* genes were retrieved from transcriptome sequences of calli of *P. koraiensis* registered in the National Center for Biotechnology Information (NCBI) sequencing read archive under accession number PRJNA880750. The effect of fungal elicitor (*P. chrysogenus* MF) on the expression levels of the *PkSTS* and *PkPMT* genes in *P. koraiensis* calli was analyzed by qPCR. The expression of *PkSTS* and *PkPMT* was weak without MF treatment but significantly enhanced by MF treatment (Figure 9A,B). Among the different times of MF treatment (0, 1, and 3 days), the highest accumulation of *PkSTS* and *PkPMT* mRNAs was detected in calli after 3 days of MF treatment (Figure 9A,B). The expression of the *PkSTS* gene was more strongly affected by MF treatment than *PkPMT* (Figure 9A,B).

### 2.7. Nematicidal Activity of Crude Extracts from P. koraiensis Calli

**Figure 10:**
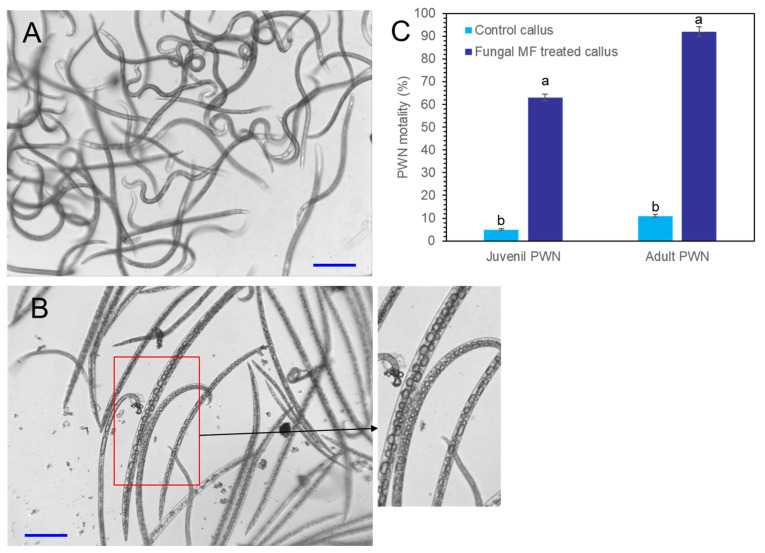
Photographs of PWN mobility and nematicidal effects of *P. koraiensis* cell extracts with and without fungal elicitor (*P. chrysogenum* MF) treatment. (**A**) PWNs with active mobility after 24 h of incubation in control *P. koraiensis* cell extracts. (**B**) PWNs with immobilized strait dead body shapes after 24 h of incubation in *P. koraiensis* cell extracts after fungal elicitor treatment for 3 days. (**C**) The concentrations of DPME and PME in *P. koraiensis* cell extracts were adjusted to 11 µg/mL and 200 µg/mL, respectively. Different letters above the bars indicate significantly different values (*p* < 0.05), calculated using one-way ANOVA followed by *Duncan’s post hoc analysis*. Scale bars = 100 μm.

Crude extracts were obtained from *P. koraiensis* cells after *P. chrysogenum* MF treatment for three days. The DPME and PME concentrations of extracts from MF-treated cells for three days were diluted to concentrations of 11 µg/mL DPME and 200 µg/mL PME, and the same dilution was also applied for the extracts from non-treated control cells. The content of DPME and PME in control cell extracts was less than 0.5 µg/mL DPME and 1.7 µg/mL PME. These crude extracts were treated to PWNs. In extracts from MF non-treated cells, most adult and juvenile PWNs showed active pendulation of their bodies until after 24 h (Figure 10A). In the PWNs treated with crude extracts from MF-treated cells, PWNs began to lose their mobility after 3 h of treatment (Figure 10B). The dead PWNs showed a strait body shape filled with highly enlarged vacuoles (Figure 10B). The extracts from MF-treated cells showed 92.5 ± 7.8% nematicidal activity for adult PWNs and 63.7 ± 3.5% nematicidal activity for juvenile PNWs. However, MF non-treated control cells showed 11.3± 1.4% nematicidal activity for adult PWNs and 5.2 ± 1.7% nematicidal activity for juvenile PWNs (Figure 10C).

## 3. Discussion

Plant cell and tissue culture are useful techniques for the production of secondary metabolites under controlled in vitro environments. Pinosylvin stilbenes have various beneficial effects such as antifungal, antibacterial, anti-inflammatory, antioxidant, neuroprotective, and anticancer activities [27]. The production of pinosylvin stilbenes was first reported by Lange et al. [22] using *P. sylvestris* cell suspension cultures. Fungal elicitor (extract from *L. seditiosum* mycelium) treatment in *P. sylvestris* cell suspensions resulted in strong accumulation of pinosylvin and PME [22]. Recently, Koo et al. [23] reported the enhanced production of pinosylvin stilbenes in *P. strobus* callus by callus aging.

*Penicillium* is commonly distributed on PWNs and their vectors and hosts [24]. It has been reported that *Penicillium* is commonly found in either PWN infected pines or heathy pines [28]. Although some fungi are toxic to PWNs [29], *Penicillium* does not show particular toxicity to PWNs [28]. Fungal elicitor treatment prepared from *Penicillium* strongly stimulated the production of both DPME and PME in cultured cells of *P. koraiensis*. The GC chromatogram of *P. koraiensis* cell extracts without fungal elicitor treatment revealed that both DPME and PME were detected in tiny peaks. The chromatogram peaks of both DPME and PME were mostly enlarged and seen as two major peaks in *P. koraiensis* cell extracts after fungal elicitor treatment. *P. chrysogenum* (KCTC 6052) fungal elicitor (MF) treatment resulted in a 97-fold increase in PME and a 10-fold increase in DPME after 3 days of treatment. This result indicates that fungal elicitor treatment is highly effective in stimulating the biosynthesis of pinosylvin stilbenes in cultured cells of *P. koraiensis*.

Although PME has the highest nematicidal activity, the existence of PME in pine plants was restricted in heartwoods [12] or knots [30]. Sapwoods, bark, and needles of pine contain no or a low amount of PME [31]. In our experiment, GC analysis revealed that the extracts of stem bark and needles of *P. koraiensis* contain very low amounts of DPME and PME. Thus, the cell culture system, coupled with fungal elicitor treatment, is very effective in producing PME.

The ratio of PME and DPME accumulation in the *P. koraiensis* cell suspension was different between the fungal medium filtrate (MF) and fungal cell extract (CE). MF treatment was effective for PME production. In contrast, CE treatment was effective both for DPME and PME accumulation, although the total amount of both PME and DPME in CE-treated cells was lower than that in MF-treated cells. This result might be caused by the chemical compositions of MF and CE, which affect the biosynthesis of PME and DPME.

Recently, Koo et al. [23] reported the enhanced production of pinosylvin stilbenes, particularly for the production of DPME, by prolonged culture of *P. strobus* calli. To investigate the effect of callus ageing on PME and DPME accumulation, *P. koraiensis* calli were sampled after 1, 2 and 3 months of culture. The browning of callus color rapidly proceeded with prolonged culture. DPME and PME were detected in small amount in non-brown calli. However, there was a small enhancement of PME during prolonged callus culture compared to *P. strobus* callus culture. This result suggests that the genes involved in the biosynthesis of pinosylvin stilbenes may be differentially regulated species, specifically under biotic and abiotic stresses. 

It has been reported that PME and DPME accumulation in branches after PWN infection differs between PWN-susceptible and PWN-resistant pines. When PWNs were inoculated in the branches of two PWN-susceptible pines (*P. koraiensis* and *P. densiflora*) and PWN-resistant pine (*P. strobus*), the two PWN-susceptible pines showed no obvious enhancement of PME and DPME accumulation in branches after PWN infection, but *P. strobus* showed a strong enhancement of PME and DPME accumulation [16]. In highly PWN-susceptible *Pinus thunbergii*, PME was maintained in trace amounts even after PWN infection [32]. It is interesting that fungal elicitor treatment strongly stimulated the production of PME and DPME in cultured cells of PWN-susceptible pine (*P. koraiensis*).

Recently, we reported the strong nematicidal activity of DPME and PME against PWNs [16,23]. We investigated the nematicidal activity of ethanolic crude extracts from *P. koraiensis* cells with and without fungal elicitor (MF) treatment. The crude extracts of *P. koraiensis* cells without MF treatment did not show nematicidal activity against PWNs. However, the crude extracts of *P. koraiensis* cells after MF treatment showed strong nematicidal activity.

## 4. Materials and Methods

### 4.1. Callus Induction from Mature Zygotic Embryos of P. koraiensis

Mature seeds of *P. koraiensis* were provided by the National Forest Seed and Variety Center in the Korea Forest service, Chungju-si, Chungcheongbuk-do, 27495, Republic of Korea. After dehulling the shells of mature seeds, inner nuts were immersed in a 70% EtOH solution, transferred into 1% NaClO for 15 min, and rinsed with sterilized water. Five zygotic embryos were plated onto a Petri dish containing 1/2 LV medium [33] supplemented with 4.4 μM mg/L 2,4-dichlorophenoxyacetic acid (2,4-D) and 2.2 μM 6-benzylaminopurine (BA). All media were supplemented with 20 g/L sucrose and 2.8 g/L Phytagel (Sigma-Aldrich, St. Louis, MO, USA) and pH was adjusted to 5.7 before autoclaving at 121 °C. All the cultures were incubated for two weeks at 25 °C in the dark.

### 4.2. Callus Proliferation

Callus was induced from zygotic embryos after subculture of callus at 3-week intervals. To investigate the effects of medium type on callus growth, calli were cultured onto 1/2 LV medium, 1/2 WV5 medium [34], or 1/2 MS medium [35] with 2.2 μM BA and 4.4 μM 2,4-D for three weeks. Medium supplementation and culture conditions are the same as callus induction mentioned above. After 3 weeks, fresh and dry weights were measured. For statistical analysis, 300 mg of callus (separated into 12 pieces) was cultured onto a Petri dish. Each experiment was conducted with three replicates. The experiment was repeated three times.

### 4.3. Cell Suspension Culture

Cell suspension culture of *P. koraiensis* was performed by shaking a culture flask containing 1/2 LV liquid medium with 4.4 μM 2,4-D and 2.2 μM BA. To investigate cell growth during the cell suspension culture, one gram of cells was transferred into three different liquid media (1/2 LV, 1/2 WV5, and 1/2 MS) with 2.2 μM BA, 2.2 μM 2,4-D and 20 g/L sucrose in a 250 mL flask containing 75 mL medium. Culture flasks were agitated at 120 rpm in the dark at 25 °C. Time-lapsed cell growth was monitored by measuring the fresh weight of cells during 15 days of culture. Cell growth was determined by the measurement of fresh weight (FW) of cells after removal of the culture medium by filtration (Whatman No. 1). Each treatment was conducted with at least three replicates. The experiment was repeated three times.

### 4.4. Preparation of Fungal Elicitors

*P. chrysogenum* (KCTC 6052)*, P. pinophilum* (KCTC 16057), and *P. roquefortii* (KCTC 6080) for fungal elicitor preparation were obtained from The Korean Collection for Type Cultures (KCTC), the Biological Resource Center (BRC) of the Korea Research Institute of Bioscience and Biotechnology (KRIBB). Fungal cultures were performed on solid potato dextrose agar (PDA) medium. The medium composition was peeled potatoes (200 g/L), dextrose (20 g/L), yeast extract (0.1 g/L), agar (20 g/L), and twin 80 (0.5%). The pH of the medium was adjusted to 5. For the suspension culture, the fungi were grown in 250 mL flasks containing 100 mL of potato dextrose broth in the dark on gyratory shakers at 110 rpm at 25 °C.

Two types of fungal elicitors, fungal cell extract (CE) and fungal medium filtrate (MF), were prepared according to the procedure described by Baldi et al. [36]. To prepare fungal CE, fungal mycelia of *P. chrysogenum* (KCTC 6052)*, P. pinophilum* (KCTC 16057), and *P. roquefortii* (KCTC 6080) were collected after 10 days of culture from the broth by filtration and washed twice with distilled water. They were then dried at 50 °C and powdered using a mortar. Approximately 1 g of cell powder was suspended in 50 mL of double-distilled water and autoclaved at 120 °C for 15 min. The supernatant obtained after centrifuging the suspension at 5000 rpm for 20 min was used as the CE. To prepare the fungal MF, the culture broth was passed through a Whatman No. 1 filter paper (Whatman, Maidstone, UK), and then the filtrated solution was filtered again for sterilization using a 0.22 µm filter and was used as the fungal MF.

### 4.5. Fungal Elicitor Treatment during Cell Suspension Culture of P. koraiensis

The effects of fungal elicitors on the production of DPME and PME in culture *P. koraiensis* cells were monitored. Total 500 mg of calli was inoculated into 200 mL Erlenmeyer flasks containing 50 mL 1/2 LV liquid medium, 20 g/L sucrose and 2.2 μM 2,4-D and 2.2 μM BA. Culture flasks were agitated at 120 rpm under dark conditions. For the elicitation treatment, the cultures were elicited with 2.0% (*v*/*v*) concentrations of CE or CF on the 10 days of culture. The control was treated with an equal amount of PDA medium instead of CF and CE solution. Cells were harvested after 0, 1, and 3 days of culture. The contents of DPME and PME were analyzed by GC/MS. The experiment was performed in triplicate and repeated three times.

### 4.6. qRT-PCR Analysis in Fungal Elicitor-Treated Calli

Total RNA was isolated from fungal elicitor treated calli and non-treated calli by the RNeasy Plant Mini Kit for qRT-PCR (Qiagen, Hilden, Germany), and converted to cDNA using M-MLV reverse transcriptase (Invitrogen, Carlsbad, CA, USA). qPCR analysis was performed using Rotor-Gene Q (Qiagen, Hilden, Germany) with a QuantiTect SYBR Green qPCR Kit (Qiagen, Hilden, Germany). The relative expression value of the genes was calculated using the 2^−ΔΔCT^ method [37]. The β-actin gene of *P. koraiensis* was used for normalization. qPCR analysis was performed with three replicates. The data are presented as the average relative quantities ± SEs. The primers for qPCR analysis used in this study are listed in Table S1.

### 4.7. GC–MS Analysis

To investigate the amount of pinosylvin stilbenes in the leaves and stem bark of *P. koraiensis*, the stem bark and leaves were collected from three independent plants. Milled powders (200 mg) of dried samples were soaked in 100% chloroform and sonicated for 30 min at 40 °C. After centrifugation, the supernatant was collected and filtered through a 0.45 μm membrane.

The contents of PME and DPME compounds in calli or cells were measured by gas chromatography–mass spectrometry (GC–MS). Calli or cells were dried at 50 °C in a drying oven. Milled powders (100 mg) of dried samples were soaked in 100% methanol (1 mL) and sonicated for 30 min at 40 °C. The supernatant after centrifugation (15,000× *g* for 10 min) of the tube was filtered using a 0.45 μm membrane to remove debris. The aliquots were analyzed by Agilent 7890A GC (Agilent Technologies, Inc, Wilmington, DE, USA) coupled to a Agilent 5975C mass spectrometer system (Agilent Technologies, Inc, Wilmington, DE, USA) with a triple-axis detector and equipped with an HP-5MS capillary column (30 m × 0.25 mm, film thickness 0.25 mm). The injection temperature was 250 °C, and the column temperature was as follows: 70 °C for 4 min, 220 °C at a rate of 5 °C min^−1^, heating at 4 °C min^−1^ up to 320 °C, and a hold at 320 °C for 5 min. The interface temperature was 300 °C, with a split/splitless injection (10:1). The temperature of the ionization chamber was 250 °C, and ionization was performed by electron impact at 70 eV. The amounts of DPME and PME were calculated by comparison to standard compounds prepared at a range of concentrations. The standards of PME and DPME were purchased from Sigma–Aldrich Co., St. Louis, MO, USA. The experiment was performed in triplicate and repeated three times.

### 4.8. Nematicidal Activity of Callus Extracts

PWNs were cultivated on potato dextrose agar (PDA) medium with *Botrytis cinerea* for 2 weeks in darkness. The PWNs were isolated with the Baermann funnel method [38]. The nematicidal activity of *P. koraiensis* cell extracts was evaluated against PWNs. Cells harvested from control and fungal elicitor treatments were dried in an oven at 50 °C for 24 h. The dried cells were ground into fine powder using a mortar and pestle. The cell powders (4 g) were placed in a 50 mL conical tube containing 100% EtOH and sonicated for 30 min at 40 °C. The supernatant was collected after centrifugation of the tube (5000× *g* for 10 min), and then evaporated to obtain the crude extract. The evaporated crude extract was first dissolved in 100% EtOH. After analysis of PME and DPME concentrations by GC/MS, the extracts were diluted with water containing 10 mg mL/mL concentration of 2-hydroxypropyl-b-cyclodextrin (HP-β-CD) which was used as an emulsifier to dissolve hydrophobic stilbenes [39]. The final tested concentrations of DPME and PME using extracts of fungal elicitor-treated cells were adjusted to 11 μg/mL and 200 μg/mL, respectively. The same dilution of control extracts were achieved with HP-β-CD solution.

PWNs harvested using the same protocol by Hwang et al. [16] were inoculated in ibidi µ-Slide angiogenesis dishes (ibidi, Munich, Germany) containing test solution and cultured for 24 h at 25 °C. Approximately 50 PWNs were incubated in each test solution. The number of PWNs immobilized was counted under a light microscope. The experiment was performed in triplicate and repeated five times.

### 4.9. Statistical Analysis

Values in all data are presented as the average relative quantities ± standard error (SE). Statistical analysis was performed using SPSS software (SPSS Science, Chicago, IL, USA). Significant differences among means were evaluated calculated using one-way ANOVA followed by *Duncan’s post hoc analysis*.

## 5. Conclusions

We developed a protocol for establishing callus and cell suspension cultures of *P. koraiensis*. The production of pinosylvin stilbene in cultured cells of *P. koraiensis* was effectively achieved by fungal elicitor treatment. The *P. koraiensis* cell extracts after fungal elicitor treatment showed high nematicidal activity against PWNs. Thus, fungal elicitor-treated *P. koraiensis* cells can be useful sources for the production of nematicidal compounds against PWNs.

## Data Availability

Not applicable.

## Supplementary Materials

The following supporting information can be downloaded at: https://www.mdpi.com/article/10.3390/plants11212933/s1, Table S1. List of primers for qRT-PCR analysis involved in *P. koraiensis* stilbene biosynthesis.
