# Supplementary material for: Production of Nematicidal Pinosylvin Stilbenes in Cell Suspension Cultures of Pinus koraiensis by Fungal Elicitation
Source: Plants (Basel). 2022 Oct 31;11(21):2933. doi: 10.3390/plants11212933 (PMC9658687; doi:10.3390/plants11212933)
Supplement: Supplementary file 1 [file plants-11-02933-s001.zip › plants-1972731-supplementary.pdf]

Table S1. List of primers for qRT-PCR analysis involved in *P. koraiensis* stilbene biosynthesis.

**Genes**	**Primers**	
PkSTS	F:ACGGTGCCTCCAGCGAAGCA	R: CCTGAGAGAGGTAGGGCTC
PkPMT	F: GCCCTTTCCCGCATTCTTTC	R: TGCTGAGATTGGTAAGCCCG
Actin	F: CTTGCTGGGCGAGATTTGAC	R: AGCTGTCTCAAGCTCCTGTTC
